# Anti-Inflammatory and Antimicrobial Effects of Herbal Formulation Called Apo-Taat Using Extended-Spectrum ß-Lactamase-Producing *Escherichia coli* Isolates

**DOI:** 10.1155/tswj/6151640

**Published:** 2025-02-03

**Authors:** Thanakan Kitnithiprapha, Sumalee Panthong, Intouch Sakpakdeejaroen, Sumalee Kondo

**Affiliations:** ^1^Department of Applied Thai Traditional Medicine, Faculty of Medicine, Thammasat University, Pathum Thani, Thailand; ^2^Department of Preclinical Sciences, Faculty of Medicine, Thammasat University, Pathum Thani, Thailand

**Keywords:** antibacterial activity, anti-inflammatory activity, *E. coli*, herbal medicine

## Abstract

Pathogens contaminate drinking water in tropical countries causing diarrheal diseases. The conventional treatment for diarrhea is antibiotics. However, overuse and misuse of antibiotics has enabled pathogens to adapt, causing global antibiotic resistance and proliferation of extended-spectrum ß-lactamase-producing *Escherichia coli* (ESBL-*E. coli*), which causes diarrhea and high levels of inflammatory cytokines. Apo-taat, consisting of equal proportions of *Phyllanthus emblica* and *Caesalpinia sappan*, has been used to treat diarrhea and bloody diarrhea. Its antibacterial activity against *E. coli* ATCC 25922 has been reported, but its inhibitory effect against ESBL-*E. coli* has yet to be documented. This study investigated the antibacterial effect of Apo-taat extract against ESBL-*E. coli* and its anti-inflammatory activity. Antibacterial activity was determined by the microtiter plate–based method. HPLC was used to determine the brazilin and gallic acid contents in Apo-taat extract. Effects of herbal extracts on nitric oxide, IL-6, and TNF-α were investigated in RAW 264.7 cells. Results were that Apo-taat extract showed MIC values against ESBL-*E. coli* in the range of 0.625 to 2.5 mg/mL. Its 50% inhibitory concentration against nitric oxide and IL-6 production was 83.96 ± 10.60 and 83.06 ± 2.07 μg/mL, respectively, and it had slight inhibition against TNF-α. These findings suggest that Apo-taat may have an antibacterial impact on ESBL-*E. coli* and anti-inflammatory activity. Furthermore, safety and clinical trials should be conducted in the future.

## 1. Introduction

Infectious pathogens cause diarrhea leading to morbidity and mortality in children and adults, especially in the tropics [1]. Symptoms include frequent watery or loose bowel movements for 24 h. When the immune system detects these pathogens, it triggers inflammatory cytokines such as tumor necrosis factor-α (TNF-α), interleukin-6 (IL-6), and nitric oxide, which induce colitis [2–5]. The pathogen most frequently isolated in newborns and travelers to tropical regions is *Escherichia coli* [6]. The prevalence of *E. coli* infection is increasing because improper consumption of antibiotics has led to microbial resistance [7, 8]. Extended-spectrum beta-lactamase (ESBL) is an enzyme produced by the Enterobacteriaceae family that can deactivate a wide range of antibiotics, including broad-spectrum oxyimino-cephalosporin, penicillin, and monobactam, but not ß-lactamase inhibitors such as sulbactam, tazobactam, and clavulanic acid. The ESBL genes, which are plasmid-mediated, can be classified into families, including *bla*TEM, *bla*SHV, *bla*CTX-M, and *bla*OXA. The TEM type contains specific amino acid residues Gly238, Glu240, Arg164, and Glu104, which can provide resistance to ampicillin, penicillin, and first-generation cephalosporins, but not to oxyimino-cephalosporins. The SHV-type and TEM-type amino acid residues are similar; specifically, Ser238 and Glu240 are altered in this type. The blaSHV family confers resistance to penicillin, tigecycline, and piperacillin, but not to oxyimino-cephalosporin. The CTX-M type, which is the predominant ESBL family, is categorized into five groups according to sequence homologies: CTX-M-1, CTX-M-2, CTX-M-8, CTX-M-9, and CTX-M-25. They prefer hydrolyzing cefotaxime rather than ceftazidime, and their activity is hindered by tazobactam. The OXA type is a diverse group that exhibits variation in amino acid sequences and can potentially break down cephalosporins and monobactams [9, 10]. Among the Gram-negative bacterial species, *E. coli* is the primary host for ESBL, and ESBL on *E. coli* is increasingly prevalent in Asia, making antibiotic treatment ineffective [11]. While microbial resistance has been increasing, a few new antibiotics have been discovered [12]. Therefore, alternative antibiotic substances are being developed to combat microbial resistance. Plants containing antimicrobial agents, such as flavonoids and tannins, are natural sources of potentially potent antibiotics [13, 14]. Apo-taat is a traditional Thai remedy for bloody diarrhea. It was recorded in King Narai's pharmacopeia during the Ayutthaya dynasty. This herbal mixture was prepared by boiling *Phyllanthus emblica* bark and *Caesalpinia sappan* wood in equal proportions [15]. The chemical constituents of *P. emblica* bark are gallic acid, phyllanthol, glycoside compounds, 16-dehydropregnenolone, betulin, periplogenin, and methyl gallate [16]. The chemical constituents of *C. sappan* are tannins, saponin, and various phenolic constituents such as protosappanin and brazilin [17, 18]. The phytochemical compounds of the 21^st^ Apo-taat remedy are tannins, phenolic acids, and flavonoids, which have antimicrobial and anti-inflammation activity [19–21]. A previous study found that an aqueous extract of the 21^st^ Apo-taat remedy inhibited Gram-positive and Gram-negative bacteria with a minimum concentration of 0.156 to 1.25 mg/mL. Its effective compounds were brazilin and gallic acid [22]. However, the activity of Apo-taat against antibiotic-resistant bacterial species and against pathogen-induced inflammation has not been reported. This study assessed aqueous 21^st^ Apo-taat remedy for the inhibition of ESBL-*E. coli* and anti-inflammatory activity.

## 2. Materials and Methods

### 2.1. Plant Materials

The heartwood of *C. sappan* was collected at Suphanburi Province, Thailand (100° 07′ 19.85″ E longitude and 14° 28′ 27.05″ N latitude). It was identified and authenticated by the herbarium of the Southern Centre of Thai Medicinal Plants, Faculty of Pharmaceutical Sciences, Prince of Songkla University, Songkhla Province, Thailand. The specimen reference number is SKP098031901. Bark of *P. emblica* was collected at Pathum Thani Province, Thailand (100° 28′ 59.99″ E longitude and 14° 02′ 60.00″ N latitude). It was identified and authenticated by the Princess Sirindhorn Plant Herbarium Building, Department of Agriculture, Kasetsart University, Phaholyothin Road, Lat Yao, Khet Chatuchak, Bangkok, Thailand. The specimen reference number is BK No.085336. All materials were collected in January 2023 (winter season).

### 2.2. Chemical and Reagents

Nutrient agar and Mueller Hinton broth were obtained from Difco, USA. Ampicillin, lipopolysaccharide, and resazurin were obtained from Sigma-Aldrich, Germany. Levofloxacin, gallic acid, phosphoric acid, and MTT were purchased from TCI, Japan. Brazilin was purchased from ChemFaces, China. Acetonitrile and dimethyl sulfoxide were purchased from RCI Labscan, Thailand. Fetal bovine serum (FBS), penicillin/streptomycin, and Dulbecco's modified Eagle's medium were acquired from Gibco, USA. We bought ELISA kits for TNF-α and IL-6 from R&D Systems in the USA.

### 2.3. Preparation of Herbal Formulation and Its Ingredient Extracts

The Apo-taat preparation was extracted using the decoction method. The dried heartwood of *C. sappan* and bark of *P. emblica* were ground into powder. 250 g of each sample was combined to make Apo-taat powder. The 500 g of Apo-taat powder was boiled in 2000 mL of water for 15 min and then filtered. The extract was re-boiled and filtered twice more. After that, the three filtered extracts were combined, evaporated, and dried in a lyophilizer. *P. emblica* and *C. sappan* were extracted using a similar method. The three extracts, Apo-taat aqueous extract (APW), *C. sappan* aqueous extract (CSW), and *P. emblica* aqueous extract (PEW), were investigated for antibacterial and anti-inflammatory activities. All extracts were stored at −20°C until use.

### 2.4. Antibacterial Activity

Clinical strains of ESBL-*E. coli* were obtained from a previous study in which the *bla*_ESBL_ gene was detected using the polymerase chain reaction with *bla*_ESBL_ as the primer [23]. In that study, there were 21 strains including SK3, SK4, SK6, SK8, SK11, SK20, SK23, SK56, SK76, SK77, SK80, SK81, SK82, SK83, SK85, SK89, SK91, SK93, SK100, SK101, and SK114. Each clinical strain of *E. coli* had different resistance gene patterns, as shown in the previous report [23]. All 21 strains of ESBL-*E. coli* were cultured together in nutrient agar and incubated at 37°C for 18–24 h. Then, the single colony was cultured in Muller Hinton broth (MHB) and incubated for 2 h at 37°C. After that, the bacteria suspension was adjusted to 0.5 McFarland using a McFarland densitometer (Liofilchem, Italy), and then, it was used as the inoculum. The antibacterial activity was evaluated using resazurin as an indicator [24]. All aqueous extracts were prepared, adjusted to 20 mg/mL in water, and filtered through a 0.22-μm sterile syringe filter (Millipore, USA). The stock solution was diluted 2-fold in MHB. All samples and bacteria suspensions were placed in triplicate 96-well plates (Corning, USA) at 50 μL/well. A negative control consisting of 50 μL/well of MHB and 50 μL/well of suspended bacteria was prepared to detect bacteria growth. Another well plate containing only MHB at 100 μL/well was prepared to detect media contamination. Then, the plates were incubated for 18–24 h at 37°C in an orbital shaker incubator [25]. After incubation, 10 μL of resazurin at a concentration of 1 mg/mL was added to each well and incubated for 3 h at 37°C. The resulting color change was monitored visually. The resazurin changed from blue to pink where the bacteria were not suppressed. The minimum concentration of extract with no change in the color of the resazurin was the minimum inhibitory concentration (MIC). Suspensions with no change in the color of resazurin were pipetted and placed in nutrient agar. Then, the plates were incubated at 37°C for 18–24 h to detect minimal bactericidal concentration (MBC) of extract [25]. MBC was the minimal concentration with no growing bacteria. Ampicillin, levofloxacin, trimethoprim, amikacin, and cefotaxime were used as positive controls. Ampicillin (100 mg/mL) and trimethoprim (50 mg/mL) stock solutions were prepared in dimethyl sulfoxide. Levofloxacin and amikacin were prepared and adjusted to 10 mg/mL in water, whereas cefotaxime was dissolved and adjusted to 20 mg/mL in water. All stock solutions were filtrated using a 0.22-μm sterile syringe filter (Millipore, USA). The antibiotic stock solutions were diluted 2-fold in MHB and assessed for MIC values.

### 2.5. Anti-Inflammatory Activity

#### 2.5.1. Cell Culture

The macrophage RAW 264.7 cell line (reference number ATCC TIB-71) was obtained from the American Type Culture Collection. The cells were cultured in complete DMEM (DMEM containing 10% FBS, 50 μg/mL streptomycin, and 50 IU/mL penicillin). The cultured cell was grown at 37°C, 5% CO_2_, and subcultured by 0.125% trypsin every 4 days.

#### 2.5.2. Cell Viability

The toxicity of extract on cells was investigated using the 3-(4, 5-dimethylthiazol-2-yl)-2,5-diphenyltetrazolium bromide tetrazolium (MTT) assay [26]. The RAW 264.7 cells (1 × 10^5^ cells/well) were seeded in a 96-well plate (Corning, USA) and incubated at 37°C with 5% CO_2_ for 24 h. After incubation, 100 μL of each test concentration was added to the wells and incubated under the same conditions for another 24 h. After that, the medium was replaced with MTT solution (0.5 mg/mL) and incubated for 2 h. The medium was then removed, and dimethyl sulfoxide was added to each well. Optical density was measured at 570 nm using a microplate reader (Thermo Fisher Scientific, USA). The cell survival rate was calculated using equation (1). Extracts with a survival rate above 70% at any concentration were considered viable for further testing:

(1) $$\begin{array}{c} \%\text{ survival}=\frac{{\text{OD}}_{\text{sample}}}{{\text{OD}}_{\text{control}}}\times 100. \end{array}$$

#### 2.5.3. Determination of LPS-Induced Nitric Oxide and Inflammatory Cytokine Production From RAW 264.7 Cells

Using the following modified method described in Tewtrakul and Itharat, the inhibitory effect of nitric oxide production from RAW 264.7 cells was assessed [27]. The RAW 264.7 cells were seeded in a 96-well plate with 1 × 10^5^ cells/well and incubated at 37°C, 5% CO_2_ for 24 h. After that, cells were replaced with 100 μL/well of lipopolysaccharide (20 ng/mL) and different concentrations of extracts (100 μL/well). The plate was incubated at 37°C, 5% CO_2_ for 24 h. Following incubation, the supernatant was gathered to measure the production of IL-6, TNF-α, and nitric oxide. The nitric oxide production detection was performed using the Griess reagent assay by adding supernatant at 100 μL/well and Griess reagent at 100 μL/well. The plate was then measured at 570 nm, and the NaNO_2_ was calculated according to the standard curve of NaNO_2_ [27]. The production of TNF-α and IL-6 was quantified utilizing an ELISA kit. The supernatant was added to an ELISA plate coated with primary antibody and incubated for 2 h. Subsequently, the plate was washed, and a secondary antibody was applied. Subsequently, the 3,3′, 5,5″-tetramethylbenzidine substrate was added and measured with a microplate reader (Thermo Fisher Scientific, USA) at a wavelength of 450 nm, according to the manufacturer's unmodified protocol [28]. The standard curve equation was utilized to determine the amounts of IL-6 and TNF-α.

### 2.6. HPLC Analysis

Gallic acid and brazilin were analyzed using high-performance liquid chromatography or HPLC (Shimadzu, Japan). The HPLC setup utilized a Phenomenex Luna 5 *μ* C18 [2] 100A analytical column (250 × 4.60 mm, 5 micron) with a C18 guard column (Phenomenex, USA). The injection volume was set to 10 μL. The mobile phase consisted of acetonitrile (A) and 0.1% phosphoric acid (B). For gradient elution, the initial conditions were set to 95% of eluent B, decreasing linearly to 85% over 0–12 min, and further to 75% at 32 min. The gradient then reached 0% of eluent B at 37 min, where it was held for 5 min. Before injecting the next sample, the mobile phase returned to its initial composition at 42.1 min and was left to stabilize for an additional 5 min. The flow rate was maintained at 0.65 mL/min, with detection wavelengths of 286 nm for brazilin and 272 nm for gallic acid [22].

#### 2.6.1. Method Validation

The analytical method was validated following the International Conference on Harmonization (ICH) recommendations concerning linearity, accuracy, precision, limit of detection (LOD), and limit of quantitation (LOQ) [29].

##### 2.6.1.1. Linearity

Gallic acid and brazilin were weighed and diluted in methanol to prepare standard solutions at 12.5, 25, 50, 100, 200, and 400 μg/mL. Each concentration was analyzed in triplicate. Calibration curves were plotted of peak area vs concentrations of gallic acid and brazilin. The ability of an analytical method to produce results that reflect the quantity of analyte in the sample is its linearity.

##### 2.6.1.2. Accuracy

The relative standard deviation (%RSD) was calculated, and accuracy was expressed as the percent recovery of the known amount of analytes added to the sample, relative to its existing concentration. The APW sample was spiked with standard brazilin and gallic acid mixtures at concentrations of 200, 100, and 50 μg/mL. Three tests were conducted using triplicate preparations of spiked samples. The percent recovery was calculated using the formula as follows:

The recovery percentage = (detected amount/spiked amount) × 100.

##### 2.6.1.3. Precision

Calculating the RSD can show the precision of a procedure by evaluating the consistency between individual test results. Standard solutions of gallic acid and brazilin in concentrations of 100, 200, and 400 μg/mL were analyzed in triplicate to assess intraday precision. Interday precision over 3 days was assessed using the same methodology.

##### 2.6.1.4. LOD and LOQ

The lowest quantity of analyte that may be found in a sample is known as the LOD, and it was calculated as LOD = 3.3*σ*/*S*, where *S* is the calibration curve's slope and *σ* is the standard deviation of the regression lines' y-intercepts. The lowest quantity of analyte that can be quantitatively identified in a sample with appropriate precision and accuracy is known as the LOQ, and it was computed as 10*σ*/*S*.

#### 2.6.2. Analysis of the Chemical Constituents of the Apo-Taat Extract and Its Ingredient

APW, CSW, and PEW were accurately weighed (10 mg) and adjusted to 10 mg/mL in methanol. The solution was subjected to ultrasonication for 5 min. The solution was filtered through a 0.45-μm nylon syringe filter and then analyzed by HPLC. The gallic acid and brazilin contents were calculated using the above calibration curve.

### 2.7. Statistical Analysis

Three independent experiments were performed in triplicate. The data were expressed as mean and standard deviation and analyzed using one-way ANOVA. A *p* value < 0.05 indicated statistical significance.

## 3. Results and Discussion

APW had the highest extraction yield (13.84%), followed by PEW (6.17%) and CSW (4.74%). All extracts were investigated for antibacterial and anti-inflammatory activities.

### 3.1. Inhibition Effect of Apo-Taat and Plant Ingredient Extracts Against ESBL-*E. coli*

The antibiotic sensitivity of each ESBL-*E. coli* strain was assessed, including their responses to various antibiotics, as shown in Table 1. All strains were resistant to ampicillin, and some strains were resistant to trimethoprim and cefotaxime. All strains were sensitive to levofloxacin and amikacin.

Subsequent results showed that APW exhibited inhibitory effects against ESBL-*E. coli*, with MIC values ranging from 0.625 to 2.5 mg/mL (Table 2). The MIC values for PEW and CSW were 2.5 -> 5 and 1.25–2.5 mg/mL, respectively. APW and PEW exhibited high sensitivity to EC 190 sk 56, with MIC values of 0.625 and 2.5 mg/mL, respectively.

APW exhibited increased antibacterial activity when combined with the two herbal components. The enhanced effect may result from a synergistic interaction between the active compounds, supporting earlier research indicating that a combination of *Origanum vulgare* and *Hypericum perforatum* had better inhibitory activities against *Staphylococcus aureus* than individual extracts [31]. Another study demonstrated significant synergistic activity between *Eucalyptus staigeriana* and *Alpinia galanga* against *E. coli* [32]. Many phytomedicines have antibacterial properties that may function by activating enzymes to boost metabolism, interfering with nuclear-level enzymatic processes, rupturing cell walls, or interfering with secondary metabolism [33]. Synergistic action combines compounds to affect one or more of these mechanisms, producing an additive or synergistic antibacterial effect [34].

Our results expressed potent in vitro antibacterial activity of APW against different resistant strains of *E. coli*. APW had the most potent inhibitory effect on EC 190 sk 56 with MIC values of 0.625 mg/mL, followed by EC sk 3, EC sk 4, EC sk 6, EC sk 11, EC sk 76, EC sk 77, EC sk 82, EC sk 83, EC sk 85, EC sk 91, and EC 490 sk 114 with MIC values of 1.25 mg/mL. A previous study showed that the ESBL-*E. coli* used in that study had different patterns of *bla*ESBL genes. For example, EC 190 sk 56 carried *bla*TEM, *bla*CTX-M, and *bla*OXA-2, while EC sk 3 and EC sk 91 carried *bla*SHV, *bla*TEM, and *bla*OXA-2 genes [23]. Notably, different gene patterns exhibit different types of antibiotic resistance [35]. The transformation of the *bla*OXA-2 gene could potentially cause hydrolysis of cephamycin, while the expression of *bla*TEM and *bla*CTX-M affect carbapenem resistance. The *bla*TEM transformation confers resistance to ampicillin, a first-generation cephalosporin, tazobactam, cefotaxime, and piperacillin/tazobactam [36, 37].

A recent study demonstrated that some plants exhibited antibacterial activity against ESBL-producing *E. coli* strains expressing the TEM gene. The growth of ESBL-producing *E. coli* was reduced by various natural plants, such as *Myrtus communis*, *Amaranthus retroflexus*, *Cyminum cuminum*, *Marrubium vulgare*, and *Peganum harmala*. *P. harmala* exhibited significant efficacy against targeted ESBL-producing *E. coli* isolates, with a MIC range of 1.25–2.5 mg/mL. High quantities of polyphenols in plants may contribute to their strong antibacterial properties [35]. The presence of polyphenols, including brazilin and gallic acid, in APW is responsible for its strong antibacterial properties [22]. APW inhibited various strains of ESBL-*E. coli*. It has been reported to inhibit standard strains such as *Pseudomonas aeruginosa*, *Salmonella* Typhi, *Shigella dysenteriae*, *S. aureus*, and *Bacillus subtilis* [22].

Plant ingredients of APW, including CSW and PEW, showed antibacterial activity against ESBL-*E. coli*. CSW was the most potent inhibitor against EC sk 3, EC 190 sk 56, EC 388 sk 100, and EC 490 sk 114 with MIC of 1.25 mg/mL, while PEW showed low inhibition of *E. coli*. In a previous study, CSW and PEW inhibited Gram-positive and Gram-negative bacteria, such as *E. coli*, *P. aeruginosa*, *S.* Typhi, *Enterobacter aerogenes*, *S. aureus*, MRSA, and *B. subtilis* [22, 38].

Different plants possess different chemical components that exhibit different antibacterial properties. The major constituents of *P. emblica* bark are gallic acid and ellagic acid [39], while those of *C. sappan* are brazilin and protosappanin B [40, 41]. Gallic acid showed a potential antibacterial effect against *Staphylococcus epidermidis, S. aureus,* and *Klebsiella pneumoniae* [42]. Furthermore, gallic acid inhibited the TetK efflux pump for antibiotic resistance in the IS-58 strain of *S. aureus* and improved the inhibition effect of tetracyclin [43]. Ellagic acid inhibited the efflux pump of methicillin-resistant *S. aureus* [44]. Brazilin, which is the main constituent of *C. sappan*, also had antibacterial activity against *E. coli* O157:H7, enteroaggregative *E. coli* strain 042, and O104:H4 serotype and showed potential inhibition of swarming motility. In addition to swarming motility, it inhibits biofilm production in *E. coli* O157:H7 and *E. coli* strain 042 [40]. Antibacterial activity of brazilin decreased DNA and protein synthesis [45].

### 3.2. Effect of APW and Its Ingredient Extracts on RAW264.7 Cell Viability

**Figure 1:**
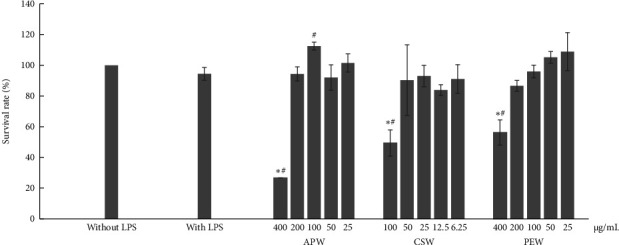
Effect of APW, CSW, and PEW on the viability of RAW264.7 cells. Error bars represent mean ± SD from three experiments. Significant differences (⁣^∗^*p* < 0.05 and ^#^*p* < 0.05) were observed when comparing to the control group without LPS treatment and to the control group with LPS treatment. APW, CSW, and PEW significantly inhibited nitric oxide and proinflammatory cytokine production.

The results showed toxicity to RAW264.7 cells at incubation with 400 μg/mL of APW, 400 μg/mL of PEW, and 100 μg/mL of CSW, as shown in Figure 1. APW and PEW concentrations of up to 200 μg/mL had no toxicity to cells, while concentrations of CSW up to 50 μg/mL were also nontoxic on RAW264.7 cells. Therefore, APW and PEW were investigated for anti-inflammatory activities with concentrations ranging from 25 to 200 μg/mL, while CSW was investigated with concentrations of 6.25, 12.5, 25, and 50 μg/mL.

**Figure 2:**
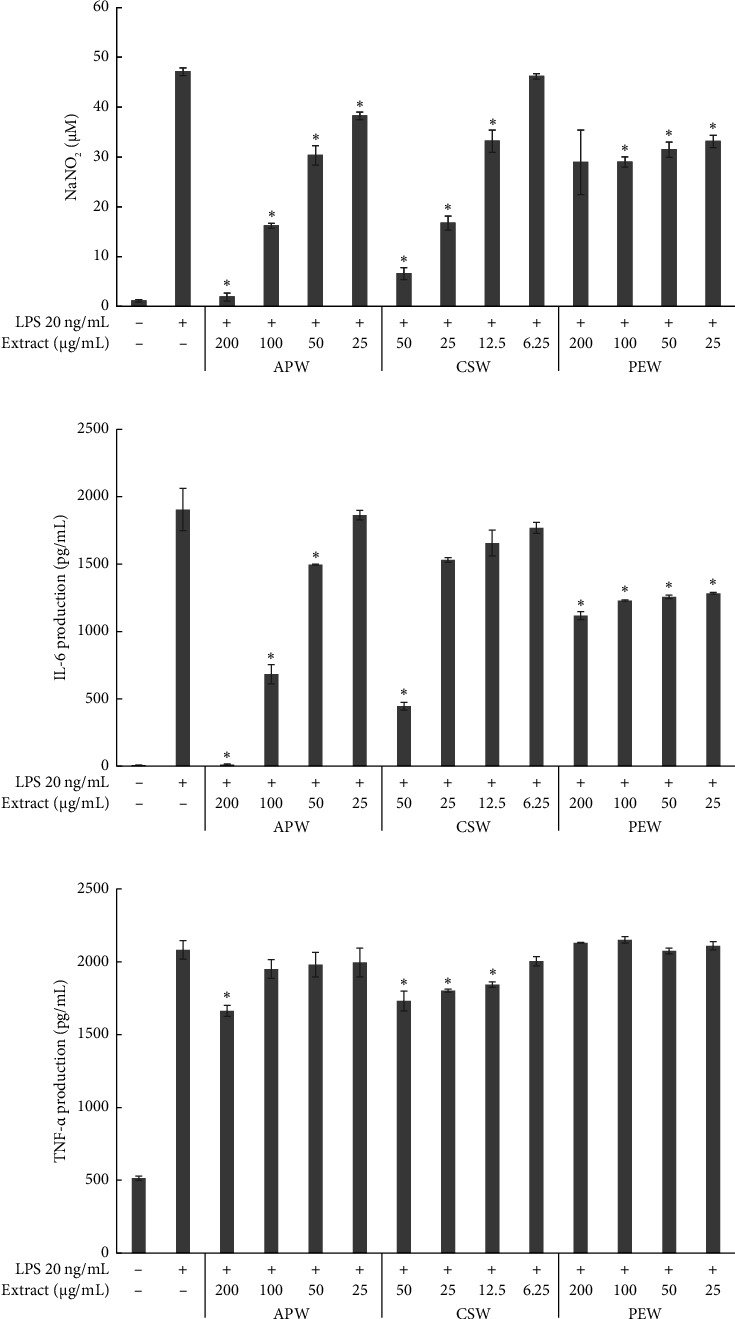
Inhibitory effect of APW, CSW, and PEW on NaNO_2_ (a), IL-6 (b), and TNF-α (c) production in LPS-stimulated RAW 264.7 cells. Error bars represent mean ± SD from three experiments. Significant differences (⁣^∗^*p* < 0.05) were observed when compared to the LPS-treated control group.

APW and CSW showed dose-dependent inhibition against nitric oxide, IL-6, and TNF-α production, while PEW markedly reduced nitric oxide and IL-6 in a dose-dependent manner. APW and CSW potently inhibited nitric oxide production with IC_50_ values of 83.96 ± 10.60 and 29.03 ± 0.38 μg/mL, respectively. APW and CSW inhibited IL-6 with IC_50_ values of 83.06 ± 2.07 and 41.88 ± 1.18 μg/mL, respectively. In contrast, the IC_50_ value of PEW was higher than the maximum concentration because the maximum inhibition was less than 50%. APW, PEW, and CSW concentrations at 100 μg/mL inhibited TNF-α production 8.15 ± 0.71, 6.42 ± 0.83, and 7.36 ± 0.33%, respectively, as shown in Figure 2.

Pathogens induce the immune response and cause inflammatory diarrhea [46]. Phagocytosis by macrophages stimulates the inflammatory response and increases the synthesis of chemokines and cytokines, which are responsible for inflammation [47]. We demonstrated for the first time that APW has a dose-dependent inhibitory effect on nitric oxide, IL-6, and TNF-α production. A similar result was found with CSW. Our findings align with a previous study reporting that CSW had high inhibition of nitric oxide and IL-6 [48]. Moreover, brazilin and sappan chalcone, constituents of *C. sappan*, have expressed almost 100% inhibition of nitric oxide production and iNOS gene expression in LPS-induced J774.1 cells [49]. Among the several cytokines and chemokines associated with immunity and inflammation, macrophages produce typical inflammatory mediators such as nitric oxide, IL-6, and TNF-α. Excessive production of these mediators might have clinical implications [50]. Phytochemicals that show anti-inflammatory activity may improve clinical symptoms and control inflammation [51, 52]. Notably, in our study, PEW showed less inhibition effect on nitric oxide production, TNF-α and IL-6. In previous research, the ethanol and methanol extract of a branch of *P. emblica* inhibited COX-2, iNOS, TNF-α, IL-1ß, and IL-6 in LPS-induced RAW 264.7 [53]. Different extraction solvents can differentially impact the biological activity of different bioactive compounds [54].

### 3.3. Validation Parameters

Linearity regression curves for gallic acid and brazilin were plotted across six concentrations. The correlation coefficients (*R*^2^) for both standard gallic acid and brazilin were close to 1, indicating strong linear relationships and a high degree of precision in the method, as shown in Table 3. The LOD and LOQ were calculated from the calibration curves, providing clear detection limits for the method. The LOD values for gallic acid and brazilin were 11.47 and 12.12 μg/mL, respectively, while the LOQ values were 34.76 and 36.75 μg/mL. Additionally, the HPLC method demonstrated precision through both intraday and interday analyses, with RSD values below 2% (Table 4). The method's accuracy was confirmed by spiking APW solutions with standards, resulting in recoveries of 100.64%–106.53% and 96.83%–109.19% for gallic acid and brazilin, respectively, as shown in Table 5.

### 3.4. The Gallic Acid and Brazilin Contents in the Apo-Taat Extract and Its Ingredients

**Figure 3:**
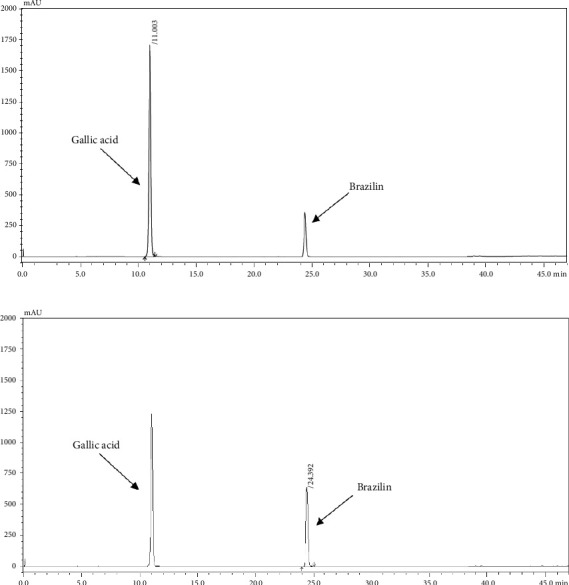
HPLC chromatogram of 400 μg/mL of gallic acid and brazilin under 272 (a) and 286 nm (b), respectively.

**Figure 4:**
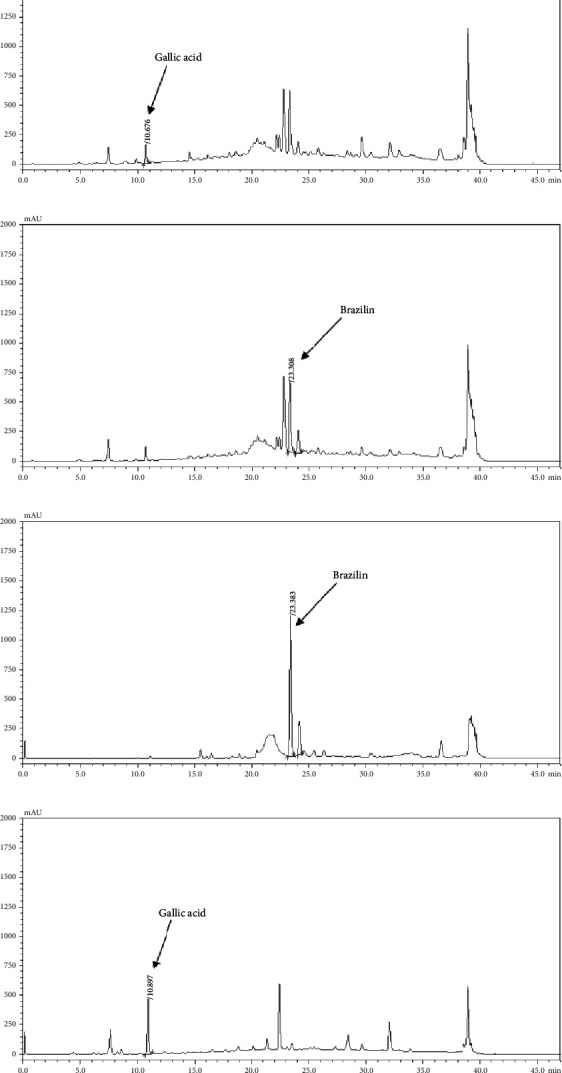
HPLC chromatogram of 10 mg/mL of Apo-taat extract under 272 (a) and 286 nm (b), respectively, 5 mg/mL of *C. sappan* extract (c) and 10 mg/mL of *P. emblica* extract (d).

The HPLC profiles of gallic acid, brazilin, Apo-taat, and its ingredients are shown in Figures 3 and 4. The major constituents of Apo-taat are brazilin (1.49% ± 0.12%w/w) and gallic acid (0.32% ± 0.01%w/w), which were found in plant ingredients. *P. emblica* consisted of gallic acid, while *C. sappan* contained brazilin. The gallic acid content in *P. emblica* extract was 0.81% ± 0.03%w/w, and the brazilin in *C. sappan* extract was 4.66% ± 0.17%w/w, as shown in Table 6. Gallic acid and brazilin have been shown to have antibacterial activity against *E. coli* and to inhibit nitric oxide and proinflammatory cytokines [48, 55]. Furthermore, gallic acid expressed bactericidal activity against clinical isolates of multidrug-resistant *E. coli* [56]. Gallic acid and brazilin may be linked with enhanced antibacterial activity and anti-inflammatory properties of APW.

## 4. Conclusions

Herbal medicine can be used as an alternate treatment for several illnesses. The Apo-taat formulation, comprising two botanical components, may serve as a possible antibacterial agent, particularly against antibiotic-resistant pathogens. Subsequent studies should evaluate the impact of the Apo-taat formulation on virulence characteristics, including toxin production, biofilm development, and its synergistic interactions with antibiotic agents. Safety and both acute and chronic toxicity of Apo-taat should also be investigated in vivo and in future clinical trial research.

## Figures and Tables

**Table 1:** The MIC values of ampicillin, levofloxacin, trimethoprim, amikacin, and cefotaxime against ESBL-*E. coli*.

Bacterial strain	Minimal inhibitory concentration (μg/mL)				
	Ampicillin	Levofloxacin	Trimethoprim	Amikacin	Cefotaxime
EC sk 3	> 1000	0.24375	> 500	6.25	1000
EC sk 4	> 1000	15.625	0.975	3.125	6.25
EC sk 6	> 1000	0.24375	> 500	3.125	2000
EC sk 8	1000	0.05	0.24375	3.125	6.25
EC sk 11	> 1000	0.4875	> 500	6.25	250
EC sk 20	> 1000	0.24375	0.24375	3.125	1000
EC sk 23	> 1000	0.24375	0.12187	3.125	1000
EC 190 sk 56	> 1000	0.4875	> 500	6.25	1000
EC sk 76	> 1000	0.025	0.24375	3.125	2000
EC sk 77	> 1000	0.05	0.24375	6.25	1000
EC sk 80	> 1000	7.8125	0.4875	6.25	2000
EC sk 81	> 1000	15.625	0.4875	12.5	5000
EC sk 82	> 1000	0.4875	> 500	6.25	2500
EC sk 83	> 1000	7.8125	> 500	6.25	10,000
EC sk 85	1000	0.05	0.4875	12.5	0.03125
EC sk 89	> 1000	7.8125	> 500	6.25	2500
EC sk 91	> 1000	15.625	> 500	12.5	10,000
EC 359 sk 93	> 1000	7.8125	> 500	6.25	1000
EC 388 sk 100	> 1000	15.625	> 500	6.25	2500
EC 410 sk 101	625	0.975	0.4875	12.5	0.0625
EC 490 sk 114	> 1000	7.8125	> 500	3.125	200

**Table 2:** The MIC (mg/mL), MBC (mg/mL), and MBC/MIC ratio values of the Apo-taat extract and its ingredients against ESBL-*E. coli*.

Bacterial strain	APW			CSW			PEW		
	MIC	MBC	MBC/MIC	MIC	MBC	MBC/MIC	MIC	MBC	MBC/MIC
EC sk 3	1.25	5	4	1.25	2.5	2	5	> 5	ND
EC sk 4	1.25	2.5	1	2.5	2.5	1	5	5	1
EC sk 6	1.25	5	1	2.5	> 5	ND	5	> 5	ND
EC sk 8	2.5	2.5	1	2.5	> 5	ND	5	> 5	ND
EC sk 11	1.25	1.25	1	2.5	> 5	ND	5	> 5	ND
EC sk 20	2.5	2.5	1	2.5	2.5	1	5	> 5	ND
EC sk 23	2.5	5	1	2.5	2.5	1	5	> 5	ND
EC 190 sk 56	0.625	0.625	1	1.25	2.5	2	2.5	> 5	ND
EC sk 76	1.25	1.25	1	2.5	2.5	1	5	> 5	ND
EC sk 77	1.25	1.25	1	2.5	2.5	1	5	> 5	ND
EC sk 80	2.5	5	1	2.5	2.5	1	5	> 5	ND
EC sk 81	2.5	2.5	1	2.5	2.5	1	5	> 5	ND
EC sk 82	1.25	5	4	2.5	2.5	1	5	> 5	ND
EC sk 83	1.25	1.25	1	2.5	2.5	1	5	5	1
EC sk 85	1.25	1.25	1	2.5	5	2	5	5	1
EC sk 89	2.5	2.5	1	2.5	2.5	1	> 5	> 5	ND
EC sk 91	1.25	2.5	2	2.5	2.5	1	> 5	> 5	ND
EC 359 sk 93	2.5	2.5	1	2.5	2.5	1	> 5	> 5	ND
EC 388 sk 100	2.5	2.5	1	1.25	2.5	2	5	5	1
EC 410 sk 101	2.5	2.5	1	2.5	2.5	1	5	5	1
EC 490 sk 114	1.25	1.25	1	1.25	1.25	1	5	> 5	ND

*Note:* MIC refers to the minimal inhibitory concentration of extract that inhibited bacteria growth, and MBC refers to the minimal bactericidal concentration of extract that killed bacteria. An MBC/MIC value ≤ 4 defined a bactericidal drug, while a value > 4 defined a bacteriostatic drug [30].

Abbreviation: ND, not detectable.

**Table 3:** HPLC validation parameters of gallic acid and brazilin.

Parameter	Gallic acid	Brazilin
Range (μg/mL)	12.5–400	12.5–400
Regression equation (*y* = *axe* + *b*)	*y* = 50587*x* − 128834	*y* = 18658*x* + 34889
*R* ^2^	0.9997	0.9997
LOD (μg/mL)	11.47	12.12
LOQ (μg/mL)	34.76	36.75

**Table 4:** The precision of gallic acid and brazilin.

Compound	Concentration (μg/mL)	Intraday^a^ (*n* = 3)		Interday^b^ (*n* = 9)	
		Measured concentration (μg/mL)	%RSD	Measured concentration (μg/mL)	%RSD
Gallic acid	100	109.33 ± 1.75	1.60	97.90 ± 1.80	1.84
	200	210.48 ± 3.09	1.46	205.22 ± 3.91	1.90
	400	427.55 ± 7.47	1.74	418.79 ± 5.65	1.35

Brazilin	100	99.01 ± 0.87	0.88	103.22 ± 1.92	1.86
	200	205.00 ± 2.48	1.20	211.52 ± 3.94	1.86
	400	398.67 ± 4.86	1.21	400.67 ± 6.63	1.65

^a^All data are expressed as mean ± SD; after doing triple analyses in a subsequent run.

^b^All data are mean ± SD, obtained by triplicate analyses per day over three different runs.

**Table 5:** Accuracy validation method of gallic acid and brazilin.

Compounds	Spiked concentration (μg/mL)	Recovery (%)			Mean ± %RSD
		N1	N2	N3	
Gallic acid	50	103.64	102.68	104.15	103.49 ± 0.72
	100	103.22	100.64	103.05	102.30 ± 1.40
	200	106.02	105.22	106.53	105.92 ± 0.62

Brazilin	50	105.01	105.73	107.45	106.06 ± 1.18
	100	99.67	96.83	99.81	98.77 ± 1.70
	200	109.19	105.78	107.72	107.56 ± 1.59

*Note:* All data are expressed as mean ± SD.

**Table 6:** Content of brazilin and gallic acid in Apo-taat extract and its constituents.

Sample	Brazilin content (%w/w)	Gallic acid content (%w/w)
APW	1.49 ± 0.12	0.32 ± 0.01
PEW	Not detectable	0.81 ± 0.03
CSW	4.66 ± 0.17	Not detectable

*Note:* All data are expressed as mean ± SD.

## Data Availability

The data used to support the findings of this study are available from the corresponding author upon reasonable request.

## References

[1] Zumla A., Ustianowski A. (2012). Tropical Diseases: Definition, Geographic Distribution, Transmission, and Classification. *Infectious Disease Clinics of North America*.

[2] Cross R. K., Wilson K. T. (2003). Nitric Oxide in Inflammatory Bowel Disease. *Inflammatory Bowel Diseases*.

[3] Priyamvada S., Gomes R., Gill R. K., Saksena S., Alrefai W. A., Dudeja P. K. (2015). Mechanisms Underlying Dysregulation of Electrolyte Absorption in Inflammatory Bowel Disease–Associated Diarrhea. *Inflammatory Bowel Diseases*.

[4] Thiagarajah J. R., Donowitz M., Verkman A. S. (2015). Secretory Diarrhoea: Mechanisms and Emerging Therapies. *Nature Reviews Gastroenterology & Hepatology*.

[5] Sattar S. B. A., Singh S. (2022). *Bacterial Gastroenteritis. StatPearls. Treasure Island (FL)*.

[6] Müller A., Bialek R., Kämper A., Fätkenheuer G., Salzberger B., Franzen C. (2001). Detection of Microsporidia in Travelers With Diarrhea. *Journal of Clinical Microbiology*.

[7] Rossolini G. M., Arena F., Pecile P., Pollini S. (2014). Update on the Antibiotic Resistance Crisis. *Current Opinion in Pharmacology*.

[8] Collignon P., Mackenzie J. S., Jeggo M., Daszak P., Richt J. A. (2013). The Importance of a One Health Approach to Preventing the Development and Spread of Antibiotic Resistance. *One Health: The Human-Animal-Environment Interfaces in Emerging Infectious Diseases: Food Safety and Security, and International and National Plans for Implementation of One Health Activities*.

[9] Castanheira M., Simner P. J., Bradford P. A. (2021). Extended-spectrum **β**-lactamases: an Update on Their Characteristics, Epidemiology and Detection. *JAC-Antimicrobial Resistance*.

[10] Husna A., Rahman M. M., Badruzzaman A. (2023). Extended-Spectrum β-Lactamases (ESBL): Challenges and Opportunities. *Biomedicines*.

[11] Salleh M. Z., Nik Zuraina N. M. N., Hajissa K., Ilias M. I., Deris Z. Z. (2022). Prevalence of Multidrug-Resistant Diarrheagenic *Escherichia coli* in Asia: A Systematic Review and Meta-Analysis. *Antibiotics*.

[12] Ventola C. L. (2015). The Antibiotic Resistance Crisis: Part 1: Causes and Threats. *P & T: A Peer-Reviewed Journal for Formulary Management*.

[13] Maugeri A., Lombardo G. E., Cirmi S. (2022). Pharmacology and Toxicology of Tannins. *Archives of Toxicology*.

[14] Gupta T., Kataria R., Sardana S. (2022). A Comprehensive Review on Current Perspectives of Flavonoids as Antimicrobial Agent. *Current Topics in Medicinal Chemistry*.

[15] Phra Vajirayana Royal Library (1917). *King Narai’s Pharmacopeia Textbook*.

[16] Khoa P. T., Quy P. B., Lien D. T. M., Phung N. K. P., Tuyet N. T. A. (2020). Chemical Study of the Stem Bark of *Phyllanthus Emblica* (Phyllanthaceae). *Vietnam Journal of Chemistry*.

[17] Saravanakumar S., Chandra J. H. (2013). Screening of Antimicrobial Activity and Phytochemical Analysis of *Caesalpinia Sappan* L. *Journal of Chemical and Pharmaceutical Research*.

[18] Zanin J. L. B., De Carvalho B. A., Salles Martineli P. (2012). The Genus Caesalpinia L.(Caesalpiniaceae): Phytochemical and Pharmacological Characteristics. *Molecules*.

[19] Prananda A. T., Dalimunthe A., Harahap U. (2023). Phyllanthus Emblica: A Comprehensive Review of Its Phytochemical Composition and Pharmacological Properties. *Frontiers in Pharmacology*.

[20] Vij T., Anil P. P., Shams R. (2023). A Comprehensive Review on Bioactive Compounds Found in Caesalpinia Sappan. *Molecules*.

[21] Fabbrini M., D’Amico F., Barone M. (2022). Polyphenol and Tannin Nutraceuticals and Their Metabolites: How the Human Gut Microbiota Influences Their Properties. *Biomolecules*.

[22] Panthong S., Sakpakdeejaroen I., Kuropakornpong P., Jaicharoensub J., Itharat A. (2020). Antibacterial Activity and Stability Evaluation of “Apo-Taat” Remedy Extract for Inhibiting Diarrhoea-Causing Bacteria. *Tropical Journal of Natural Product Research (TJNPR).*.

[23] Kondo S., Apisarnthanarak A., Trakulsomboon S. (2022). Prevalence of Extended-Spectrum β-Lactamase-producing Enterobacterales and Distribution of blaESBL Genes From Patients Who Underwent Abdominal Surgery. *Science & Technology Asia*.

[24] Sarker S. D., Nahar L., Kumarasamy Y. (2007). Microtitre Plate-Based Antibacterial Assay Incorporating Resazurin as an Indicator of Cell Growth, and its Application in the In Vitro Antibacterial Screening of Phytochemicals. *Methods*.

[25] National Committee for Clinical Laboratory Standards (1999). Methods for Determining Bactericidal Activity of Antimicrobial Agents. Approved Guideline. Document M26-A. *National Committee for Clinical Laboratory Standards*.

[26] Wallin R. F., Arscott E. (1998). A Practical Guide to ISO 10993-5: Cytotoxicity. *Medical Device and Diagnostic Industry*.

[27] Tewtrakul S., Itharat A. (2007). Nitric Oxide Inhibitory Substances From the Rhizomes of *Dioscorea Membranacea*. *Journal of Ethnopharmacology*.

[28] DuoSet Elisa Development System, R&D Systems [Internet]. https://resources.rndsystems.com/pdfs/datasheets/dy406.pdf?v=20241004&_ga=2.254611784.1732316683.1728281326-143464738.1728281326&_gac=1.149993924.1728281613.Cj0KCQjw6oi4BhD1ARIsAL6pox1trSuOUp_WJjRYpc12Ci1JhMzPDJ6B2B2infC0UCy_0PWYFasC5dIaAqZCEALw_wcB.

[29] Borman P., Elder D. (2017). Q2(R1) Validation of Analytical Procedures. *ICH Quality Guidelines*.

[30] Wald-Dickler N., Holtom P., Spellberg B. (2018). Busting the Myth of “Static vs Cidal”: A Systemic Literature Review. *Clinical Infectious Diseases*.

[31] Bahmani M., Taherikalani M., Khaksarian M. (2019). The Synergistic Effect of Hydroalcoholic Extracts of Origanum Vulgare, *Hypericum perforatum* and Their Active Components Carvacrol and Hypericin against *Staphylococcus aureus*. *Future Sci OA*.

[32] Weerakkody N. S., Caffin N., Lambert L. K., Turner M. S., Dykes G. A. (2011). Synergistic Antimicrobial Activity of Galangal (Alpinia Galanga), Rosemary (Rosmarinus Officinalis) and Lemon Iron Bark (Eucalyptus Staigerana) Extracts. *Journal of the Science of Food and Agriculture*.

[33] Cowan M. M. (1999). Plant Products as Antimicrobial Agents. *Clinical Microbiology Reviews*.

[34] Adwan G. M., Abu-Shanab B., Adwan K., Abu-Shanab F. (2006). Antibacterial Effects of Nutraceutical Plants Growing in Palestine on *Pseudomonas aeruginosa*. *Turkish Journal of Biology*.

[35] Lien L., Lan P. T., Chuc N. T. K. (2017). Antibiotic Resistance and Antibiotic Resistance Genes in *Escherichia coli* Isolates from Hospital Wastewater in Vietnam. *International Journal of Environmental Research and Public Health*.

[36] Nathisuwan S., Burgess D. S., Lewis J. S. (2001). Extended‐Spectrum β‐Lactamases: Epidemiology, Detection, and Treatment. *Pharmacotherapy: The Journal of Human Pharmacology and Drug Therapy*.

[37] Bradford P. A. (2001). Extended-Spectrum β-Lactamases in the 21st Century: Characterization, Epidemiology, and Detection of This Important Resistance Threat. *Clinical Microbiology Reviews*.

[38] Kumar A. T. B., Rahiman S., Gupta U. (2011). Comparative Study of Antimicrobial Activity and Phytochemical Analysis of Methanolic and Aqueous Extracts of the Fruit of *Emblica Officinalis* against Pathogenic Bacteria. *Traditional Chinese Medicine*.

[39] Chaphalkar R., Apte K. G., Talekar Y., Ojha S. K., Nandave M. (2017). Antioxidants of *Phyllanthus Emblica* L. Bark Extract Provide Hepatoprotection against Ethanol-Induced Hepatic Damage: A Comparison with Silymarin. *Oxidative Medicine and Cellular Longevity*.

[40] García-Heredia A., García S., Merino-Mascorro J. Á, Feng P., Heredia N. (2016). Natural Plant Products Inhibits Growth and Alters the Swarming Motility, Biofilm Formation, and Expression of Virulence Genes in Enteroaggregative and Enterohemorrhagic *Escherichia coli*. *Food microbiology*.

[41] Gondokesumo M. E., Kurniawan I. M. (2020). Molecular Docking Study of Sappan Wood Extract to Inhibit PBP2A Enzyme on Methicillin-Resistant *Staphylococcus aureus* (MRSA). *Journal of Basic and Clinical Physiology and Pharmacology*.

[42] Pinho E., Ferreira I. C., Barros L., Carvalho A. M., Soares G., Henriques M. (2014). Antibacterial Potential of Northeastern Portugal Wild Plant Extracts and Respective Phenolic Compounds. *BioMed Research International*.

[43] Macêdo N. S., Barbosa C. RdS., Bezerra A. H. (2022). Evaluation of Ellagic Acid and Gallic Acid as Efflux Pump Inhibitors in Strains of *Staphylococcus aureus*. *Biology Open*.

[44] AL Al Meani S. (2021). Antibacterial Activity of Ellagic Acid Against Methicillin Resistant *staphylococcus Aureus* Planktonic Cells and Biofilm.

[45] Nirmal N. P., Rajput M. S., Prasad R. G., Ahmad M. (2015). Brazilin from *Caesalpinia Sappan* Heartwood and Its Pharmacological Activities: A Review. *Asian Pacific Journal of Tropical Medicine*.

[46] Navaneethan U., Giannella R. A. (2008). Mechanisms of Infectious Diarrhea. *Nature Clinical Practice Gastroenterology & Hepatology*.

[47] Ibrahim I. B. M., Pidaparti R. (2019). Influence of Pathogens and Mechanical Stimuli in Inflammation. *Bioengineering*.

[48] Pattananandecha T., Apichai S., Julsrigival J., Ogata F., Kawasaki N., Saenjum C. (2022). Antibacterial Activity Against Foodborne Pathogens and Inhibitory Effect on Anti-inflammatory Mediators’ Production of Brazilin-Enriched Extract From *Caesalpinia Sappan* Linn. *Plants*.

[49] Sasaki Y., Hosokawa T., Nagai M., Nagumo S. (2007). In Vitro Study for Inhibition of NO Production about Constituents of Sappan Lignum. *Biological and Pharmaceutical Bulletin*.

[50] Soufli I., Toumi R., Rafa H., Touil-Boukoffa C. (2016). Overview of Cytokines and Nitric Oxide Involvement in Immuno-Pathogenesis of Inflammatory Bowel Diseases. *World Journal of Gastrointestinal Pharmacology and Therapeutics*.

[51] Pinna G. F., Fiorucci M., Reimund J. M., Taquet N., Arondel Y., Muller C. D. (2004). Celastrol Inhibits Pro-inflammatory Cytokine Secretion in Crohn’s Disease Biopsies. *Biochemical and Biophysical Research Communications*.

[52] Dong L., Du H., Zhang M. (2022). Anti-inflammatory Effect of Rhein on Ulcerative Colitis via Inhibiting PI3K/Akt/mTOR Signaling Pathway and Regulating Gut Microbiota. *Phytotherapy Research*.

[53] Sripanidkulchai B., Junlatat J. (2014). Bioactivities of Alcohol Based Extracts of *Phyllanthus Emblica* Branches: Antioxidation, Antimelanogenesis and Anti-inflammation. *Journal of Natural Medicines*.

[54] Ahmed S. A., Shaker S. E., Shawky H. (2022). Solvent Polarity Dictates the Anti-inflammatory Potency and Mechanism of Two Purslane (Portulaca Oleracea) Seed Extracts. *Journal of Food Biochemistry*.

[55] Kang J., Li Q., Liu L., Jin W., Wang J., Sun Y. (2018). The Specific Effect of Gallic Acid on Escherichia coli Biofilm Formation by Regulating pgaABCD Genes Expression. *Applied Microbiology and Biotechnology*.

[56] Tian Q., Wei S., Su H. (2022). Bactericidal Activity of Gallic Acid against Multi-Drug Resistance *Escherichia coli*. *Microbial Pathogenesis*.

